# Hypoxic pulmonary endothelial cells release epidermal growth factor leading to vascular smooth muscle cell arginase‐2 expression and proliferation

**DOI:** 10.14814/phy2.15342

**Published:** 2022-06-08

**Authors:** Bernadette Chen, Yi Jin, Caitlyn M. Pool, Yusen Liu, Leif D. Nelin

**Affiliations:** ^1^ Pulmonary Hypertension Group Center for Perinatal Research Abigail Wexner Research Institute at Nationwide Children’s Hospital and Department of Pediatrics The Ohio State University Columbus Ohio USA

**Keywords:** epidermal growth factor receptor, pulmonary hypertension, pulmonary vascular remodeling

## Abstract

The hallmark of pulmonary hypertension (PH) is vascular remodeling. We have previously shown that human pulmonary microvascular endothelial cells (hPMVEC) respond to hypoxia with epidermal growth factor (EGF) mediated activation of the receptor tyrosine kinase, EGF receptor (EGFR), resulting in arginase‐2 (Arg2)‐dependent proliferation. We hypothesized that the release of EGF by hPMVEC could result in the proliferation of human pulmonary arterial smooth muscle cells (hPASMC) via activation of EGFR on the hPASMC leading to Arg2 up‐regulation. To test this hypothesis, we used conditioned media (CM) from hPMVEC grown either in normoxia (NCM) or hypoxia (HCM). Human PASMC were incubated in normoxia with either HCM or NCM, and HCM caused significant induction of Arg2 and viable cell numbers. When HCM was generated with either an EGF‐neutralizing antibody or an EGFR blocking antibody the resulting HCM did not induce Arg2 or increase viable cell numbers in hPASMC. Adding an EGFR blocking antibody to HCM, prevented the HCM‐induced increase in Arg2 and viable cell numbers. HCM induced robust phosphorylation of hPASMC EGFR. When hPASMC were transfected with siRNA against EGFR the HCM‐induced increase in viable cell numbers was prevented. When hPASMC were treated with the arginase antagonist nor‐NOHA, the HCM‐induced increase in viable cell numbers was prevented. These data suggest that hypoxic hPMVEC releases EGF, which activates hPASMC EGFR leading to Arg2 protein expression and an increase in viable cell numbers. We speculate that EGF neutralizing antibodies or EGFR blocking antibodies represent potential therapeutics to prevent and/or attenuate vascular remodeling in PH associated with hypoxia.

## Graphical abstract

We hypothesized that the release of epidermal growth factor (EGF) by human pulmonary microvascular endothelial cells (hPMVEC) could result in the proliferation of human pulmonary arterial smooth muscle cells via activation of the EGF receptor (EGFR) on the hPASMC leading to Arg2 up‐regulation. Our data suggest that hypoxic hPMVEC do release EGF, which activates hPASMC EGFR leading to Arg2 protein expression and an increase in viable smooth muscle cell numbers.

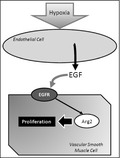

## 1 INTRODUCTION

Vasoconstriction and vascular remodeling in the pulmonary circulation are the hallmarks of pulmonary hypertension (PH). Although PH is a disease with a wide variety of etiologies, PH is associated with conditions that include a component of hypoxia, such as chronic lung diseases and high altitude (Simonneau et al., 2019). The WHO classification system groups the multitude of etiologies of PH into five broad groups, where Group 1 is pulmonary arterial hypertension (PAH) and Group 3 is PH due to lung diseases and/or hypoxia (Simonneau et al., 2019). Our laboratory has a particular interest in Group 3 PH, specifically the PH associated with bronchopulmonary dysplasia (BPD). There are currently no available therapies specifically targeting the abnormal vascular remodeling that underlies the pathogenesis of PH. The PH‐associated abnormal vascular remodeling depends on cellular proliferation in the arterial wall (Huetsch et al., 2019; Kearney et al., 2021). Although endothelial cells can proliferate contributing to this cellular proliferation, perhaps a more important role of endothelial cells in vascular remodeling is the release of factors that induce proliferation in the underlying smooth muscle cells (SMCs) leading to the intimal thickening characteristic of PH (Kurakula et al., 2021).

We have shown in both human pulmonary microvascular endothelial cells (hPMVEC) (Toby et al., 2010; White et al., 2017) and in human pulmonary artery SMS (hPASMC) (Chen et al., 2009; Xue et al., 2017) incubated in hypoxic conditions that hypoxia‐induced cellular proliferation depends on arginase‐2 (Arg2). Arg2 catalyzes the hydrolysis of L‐arginine to L‐ornithine and urea (Trittmann et al., 2018). L‐ornithine can then be further metabolized to polyamines and proline, which are essential for cellular proliferation (Caldwell et al., 2018). We have previously shown that in cultured hPMVEC the hypoxic induction of Arg2 depends on activation of the receptor tyrosine kinase, epidermal growth factor receptor (EGFR) (Toby et al., 2010). EGFR has a number of ligands, and the archetypical ligand is epidermal growth factor (EGF). Furthermore, we have described that EGFR activation on hPMVEC leads to the downstream activation of extracellular signal‐regulated kinase (ERK) and that this EGFR‐ERK pathway is necessary for hypoxia‐induced Arg2 expression in hPMVEC (White et al., 2017). Recently, we demonstrated in hPMVEC incubated in hypoxic conditions that EGF‐mediated activation of EGFR was necessary for hypoxia‐induced Arg2 expression and proliferation (Pool et al., 2018). Furthermore, we demonstrated that conditioned media from hypoxic hPMVEC induced proliferation in hPASMC that was prevented by treating the hPMVEC with an siRNA against EGF prior to generating the hypoxic conditioned media (see figure 8 in Pool et al. (2018)). These data support our postulate that hypoxia induces EGF production in pulmonary endothelial cells, which is released and can cause proliferation in vascular SMCs. Therefore, in this study we directly tested the hypothesis that the release of EGF by hypoxic hPMVEC would result in activation of EGFR on hPASMC which would lead to up‐regulation of hPASMC Arg2 protein and proliferation of hPASMC. We used conditioned media from hPMVEC to address this hypothesis. An antibody that binds with EGF to prevent EGF binding to surface receptors was used, which we designated an EGF neutralizing antibody (EGFnAb). An antibody that binds with EGFR to prevent ligand binding was used, which we designated an EGFR blocking antibody (EGFRbAb). We knocked down EGFR expression in hPASMC using transfection with siRNA against EGFR. Finally, we inhibited arginase activity using a small molecule arginase antagonist, N^ω^‐hydroxy‐nor‐L‐arginine dihydrochloride (nor‐NOHA).

## 2 MATERIALS AND METHODS

### 2.1 Generating conditioned media from human pulmonary microvascular endothelial cell (hPMVEC) cultures

Human PMVEC were cultured as previously described (Pool et al., 2018; Toby et al., 2010; White et al., 2017). Briefly, fetal hPMVEC were purchased from ScienCell (Carlsbad, CA) and were all from females including lot#5016, lot#15900, lot#17807, and lot#15902. The hPMVEC were cultured in endothelial cell (EC) media (ScienCell) supplemented with an EC media kit containing fetal bovine serum, endothelial cell growth supplement, and a penicillin/streptomycin solution (ScienCell). Human PMVEC between passages 4 and 7 were used to generate cultured media. The hPMVEC were incubated at 37°C in either 5% CO_2_, balance air (normoxia) or 5% CO_2_, 1% O_2_, balance N_2_ (hypoxia) for 24 h, when the media was harvested for the conditioned media experiments. The media from the hPVMEC incubated in normoxia was termed normoxic conditioned media (NCM) and the media from the hPMVEC incubated in hypoxia was termed hypoxic conditioned media (HCM). For some experiments, 1 µg/mL of EGF neutralizing antibody (anti‐hEGF; R&D Systems, Minneapolis, MN) or an IgG control antibody was added to the EC media prior to generating HCM (EGFnAb‐HCM or IgG‐HCM). For other experiments, 20 μg/mL of EGF receptor blocking antibody (anti‐EGFR; EMD Millipore, Temecula, CA) or an IgG control antibody were added to the EC media prior to generating HCM (EGFRbAb‐HCM or IgG‐HCM).

### 2.2 Human pulmonary artery smooth muscle cell (hPASMC) culture

Human PASMC were cultured as previously described (Chen et al., 2009; Xue et al., 2017). Briefly, fetal hPASMC were purchased from ScienCell, were all from females and included lot#7449 and lot#4551. The hPASMC were cultured in SMC media (ScienCell, Carlsbad, CA) supplemented with a SMC kit containing fetal bovine serum, SMC growth supplement, and a penicillin/streptomycin solution (ScienCell). Human PASMC between passages 4 and 8 were used for these studies. For the experiments, the NCM or HCM was mixed with SMC media such that there were 50% conditioned media from the hPMVEC and 50% SMC media as previously described (Pool et al., 2018). For some experiments, the HCM had 20 μg/mL of EGF receptor blocking antibody (anti‐EGFR; EMD Millipore, Temecula, CA) added prior to placement on the hPASMC (HCM + EGFRbAb). For all experiments, hPASMC were incubated at 37°C in 5% CO_2_, balance air (normoxia) for 24 h and cells were harvested for protein extraction.

For the siRNA experiments, hPASMC were treated with vehicle, scramble siRNA (100 nM), or siRNA against EGFR (100 nM; Silencer Select ID no. s563, catalog no. 4390824, Invitrogen, Waltham, MA) using Dharmafect (Dharmacon Inc., Lafayette, CO) transfection reagent for 24 h, as we have described previously (Xue et al., 2017). The hPASMC were washed with Dulbecco’s Phosphate‐Buffered Saline (DPBS; Corning Inc., Corning, NY) and allowed to recover in normoxia for 24 h prior to the study.

### 2.3 Protein isolation

Protein was isolated from hPASMC as previously described (Chen et al., 2009; Pool et al., 2018; White et al., 2017). Briefly, hPASMC were washed with DPBS and 50 μl lysis buffer (20 mM HEPES (pH 7.4), 40 mM β‐glycerophosphate, 2 mM EGTA, 1 mM DTT, 10 mM NaF, 1 mM Na3VO4, 1% Triton X‐100, and 10% glycerol) was added. Thirty minutes before use, the following protease inhibitors were added to each milliliter of lysis buffer: 1 μg aprotinin, 1 μg leupeptin, 1 μg pepstatin A, and 1 μg phenylmethylsulfonyl fluoride. The hPASMC were scraped and placed in sterile centrifuge tubes on ice. The samples were centrifuged at 14,000 RPM for 15 min at 4°C. The supernatant was stored at −80°C for subsequent Western blot analysis. Total protein concentration was determined by the Bradford method using a commercially available assay (BioRad, Hercules, CA).

### 2.4 Immunoblotting

Cell lysates were assayed for Arg2, phosphorylated EGFR (pEGFR), total EGFR, and β‐actin using Western blot analysis as previously described (Toby et al., 2010). Aliquots of cell lysate were diluted with appropriate amounts of 10x NuPAGE reducing agent, 4× NuPAGE LDS sample buffer, and deionized water. The samples were then heated to 80°C for 10 min, and then separated using sodium dodecyl sulfate‐polyacrylamide gel electrophoresis (SDS‐PAGE). The proteins were transferred to polyvinylidene difluoride membranes and blocked in Tris‐buffered saline with 0.1% Tween (TBS‐T) containing 10% skim milk for 1 h. The membranes were then washed with TBS‐T and incubated with primary antibody against Arg2 (1:500; Santa Cruz Biotechnology, Dallas, TX, catalogue #sc‐20151, lot# A2512), pEGFR (Tyr1148; 1:1000; Cell Signaling, Danvers, MA; catalogue #4404, lot #3), or EGFR (D38B1; 1:1000; Cell Signaling, catalog #4267, lot #11) for 1 h. The membranes were then washed three times with TBS‐T and incubated with goat anti‐rabbit IgG horseradish peroxidase (HRP) conjugated secondary antibody (1:10,000; Bio‐Rad) for 1 h. Then the membranes were washed three times with TBS‐T. The bands were visualized using chemiluminescence (Amersham ECL, Piscataway, NJ) and quantified using densitometry (Total Lab gel analysis; Biosystematica). To control for protein loading, the blots were stripped using a stripping buffer (62.5 mM Tris‐HCl pH 6.8, 2% SDS, 100 mM β‐mercaptoethanol) and re‐probed for β‐actin using a monoclonal antibody (1:10,000; Sigma, Catalog #A1978‐200UL, control #010M4816). For the pEGFR experiments, protein loading was also controlled for by stripping and re‐probing for total EGFR (D38B1; 1:1000; Cell Signaling, catalog #4267, lot #11).

### 2.5 Trypan blue exclusion for determining viable cell numbers

Viable cell numbers were determined as previously reported (Pool et al., 2018; White et al., 2017). The same number of cultured hPASMC were seeded in each well of 6‐well plates. The appropriate treatments were included in the conditioned media and the hPASMC were placed in normoxia for 48 h. The plates were then washed twice with DPBS and 1 ml of trypsin was added to each well. The plates were incubated for 3 minutes followed by the addition of 2 ml trypsin neutralizing solution. The cells from each well were placed in 15 ml conical tubes. The cells were centrifuged for 5 min at 1220 × *g* at 4°C. The supernatant was discarded and the cells were resuspended in 1 ml of EC media. The cells were mixed 1:1 with trypan blue and viable cells were counted using a hemocytometer.

### 2.6 Statistical analysis

Data are shown in the figures as median with intraquartile range and 5 to 95% confidence intervals. A *t* test was used to compare data between two groups and a one‐way analysis of variance (ANOVA) was used to compare the data between more than two groups. For the one‐way ANOVA, significant differences were identified using a Student‐Newman‐Keuls post‐hoc test (SigmaStat 14.0, Jandel Scientific, Carlsbad, CA). Differences were considered significant when *p* < 0.05.

## 3 RESULTS

### 3.1 Hypoxic conditioned media from hPMVEC resulted in Arg2 protein induction and proliferation in hPASMC

**FIGURE 1:**
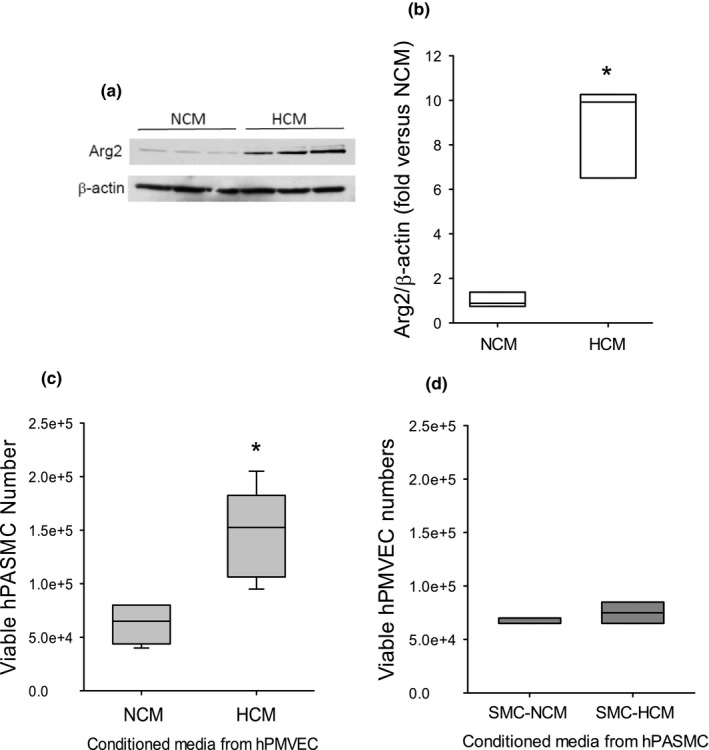
Conditioned media from hPMVEC incubated in hypoxia induced Arg2 protein expression and proliferation in normoxic hPASMC. Conditioned media was generated from hPMVEC incubated in normoxia (NCM) or hypoxia (HCM) for 24 h. The NCM or HCM was then placed on hPASMC with smooth muscle media in a 50:50 ratio and the hPASMC were incubated in room air with 5% CO_2_ for 24 h. (a) Protein was harvested for western blotting for Arg2 and β‐actin. Representative western blot images. (b) Arg2 protein levels were greater in hPASMC incubated with HCM than in those incubated with NCM. Densitometry for the Arg2 western blots normalized to β‐actin. *Different from NCM *p* < 0.003. *n* = 3 for each group. (c) To measure viable hPASMC numbers, 1 × 10^4^ hPASMC were plated in each well of 6‐well plates, NCM or HCM were placed on the cells, and after incubating in normoxia for 48 h viable cell numbers were determined using trypan blue exclusion. Viable hPASMC numbers were significantly greater after incubation with HCM than after incubation with NCM. *Ddifferent from NCM *p* < 0.0001. *n* = 6 for each group. (d) To determine if conditioned media from hypoxic hPASMC would stimulate an increase in hPMVEC viable cell numbers, conditioned media was generated from hPASMC incubated for 24 h in either 20% O_2_/5% CO_2_ (SMC‐NCM) or 1% O_2_/5% CO_2_ (SMC‐HCM). Human PMVEC (1 × 10^4^) were plated in each well of 6 well plates, the SMC‐NCM or SMC‐HCM were placed on the hPMVEC, and after 48 h viable cell number determined using trypan blue exclusion. There was no difference (*p* = 0.33) in viable endothelial cell numbers between hPMVEC incubated in SMC‐HCM versus hPMVEC incubated in SMC‐NCM. *n* = 6 for each group.

To determine if HCM would induce Arg2 protein expression in hPASMC incubated in normoxia, western blot analysis of cell lysates was used to detect Arg2 in hPASMC incubated with either NCM or HCM. In hPASMC treated with NCM and incubated in normoxia for 24 h, there were detectable Arg2 bands on western blots (Figure 1a). Whereas hPASMC treated with HCM in normoxia for 24 h resulted in significantly greater Arg2 protein levels than in hPASMC treated with NCM (Figure 1a,b). To determine if HCM would result in greater viable cell numbers, 1 × 10^4^ hPASMC were seeded in each well of 6‐well plates, treated with either NCM or HCM for 48 h, and then viable cells determined. Human PASMC treated with NCM and incubated in normoxia had ~0.6 × 10^5^ viable cells after 48 h (Figure 1c), while hPASMC treated with HCM and incubated in normoxia for 48 h had ~2.5‐fold more viable cells than hPASMC treated with NCM (Figure 1c).

To determine if the “reverse” experiment, that is, if conditioned media from SMCs incubated in hypoxia placed on endothelial cells, would cause endothelial cell proliferation (i.e., a greater number of viable hPMVEC). Human PASMC were incubated in either hypoxia (1% O_2_/5% CO_2_/balance N_2_; SMC‐HCM) or normoxia (5% CO_2_/balance air; SMC‐NCM) for 24 h to generate the SMC conditioned media. Either SMC‐HCM or SMC‐NCM was added to endothelial cell media such that 50% was conditioned media and 50% was endothelial cell media. Human PMVEC (1 × 10^4^) were seeded into each well on 6‐well plates, SMC‐HCM or SMC‐NCM was placed on the hPMVEC and then incubated for 48 h and viable endothelial cell numbers were determined. The number of viable hPMVECs were not different between the SMC‐NCM and SMC‐HCM treated cells (Figure 1d).

### 3.2 The role of EGF in the HCM‐induced increase in Arg2 protein expression and viable cell numbers in hPASMC

**FIGURE 2:**
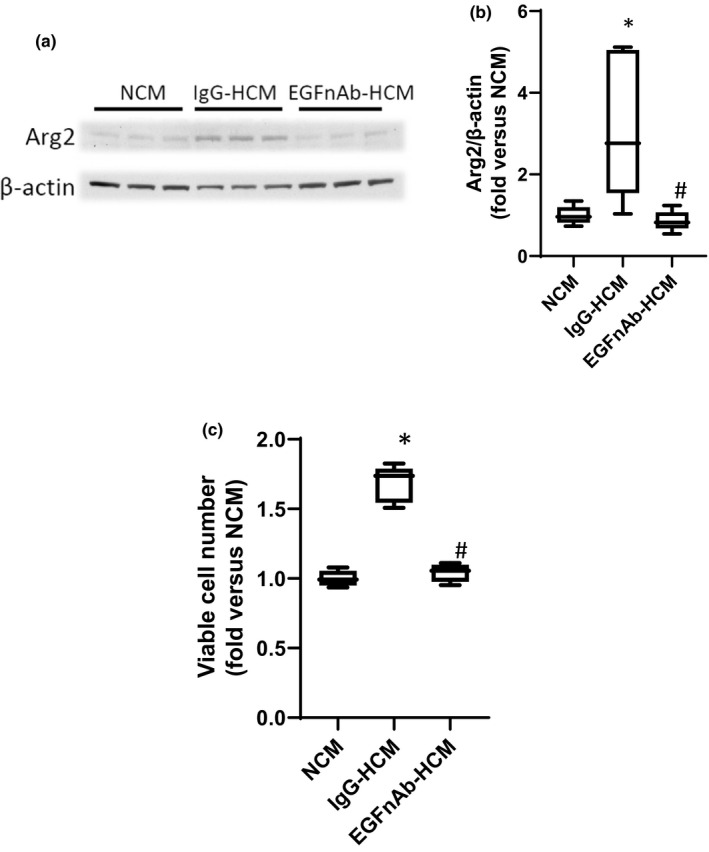
Treatment with HCM generated with an antibody against EGF prevented the HCM‐induced increases in hPASMC Arg2 protein and viable cell numbers. HCM was generated from hPMVEC treated with either an IgG control (IgG‐HCM) or an antibody against EGF (EGFnAb‐HCM), NCM was also generated as described in Figure 1. The NCM, IgG‐HCM, or EGFnAb‐HCM was then placed on hPASMC with smooth muscle media in a 50:50 ratio and the hPASMC were incubated in room air with 5% CO_2_ for 24 h. (a) Protein was harvested for western blotting for Arg2 and β‐actin. Representative western blot images. (b) Arg2 protein levels were greater in hPASMC incubated with IgG‐HCM than in those incubated with EGFnAb‐HCM, and there was no difference between Arg2 protein levels from hPASMC incubated in NCM or EGFnAb‐HCM. Densitometry for the Arg2 western blots normalized to β‐actin. * different from NCM *p* < 0.001. # EGFnAb‐HCM different from IgG‐HCM, *p* < 0.01. *n* = 6 for each group. (c) To measure viable hPASMC numbers, 1 × 10^4^ hPASMC were plated in each well of 6 well plates, NCM, IgG‐HCM, or EGFnAb‐HCM were placed on the cells, and after incubating for 48 h in normoxia viable cell number determined using trypan blue exclusion. Viable hPASMC numbers were significantly greater after incubation with IgG‐HCM than after incubation with EGFnAb‐HCM, and there was no difference in viable cell numbers between hPASMC incubated in NCM versus those incubated in EGFnAb‐HCM. *Different from NCM *p* < 0.001, # EGFnAb‐HCM different from HCM, *p* < 0.001. *n* = 6 for each group.

We have previously shown (figure 8 in Pool et al. (2018)) that HCM‐induced increases in hPASMC Arg2 and proliferation were prevented using EGF‐deficient conditioned media collected after transfection with siRNA against EGF in hPMVEC that were placed in hypoxia for 24 h. To further delineate the role of released EGF into the conditioned media on the HCM‐induced increases in Arg2 protein expression and viable cell numbers we used an EGF neutralizing antibody added to the EC media during the generation of conditioned media. The hPMVEC were treated with either EGF neutralizing antibody (EGFnAb) or an isotype IgG control antibody (IgG) during incubation in hypoxia for 24 h to generate either EGFnAb‐HCM or IgG‐HCM. Untreated hPMVEC incubated in normoxia were used to generate NCM. Human PASMC were then incubated with either NCM, IgG‐HCM, or EGFnAb‐HCM in normoxia for 24 h. Protein was harvested for Arg2 western blots. When the hPASMC were incubated with EGFnAb‐HCM the levels of Arg2 protein were not different from cells incubated with NCM and were significantly lower than Arg2 protein levels in the hPASMC incubated with IgG‐HCM (Figure 2a,b). To determine the effect of the EGFnAb on viable cell numbers, 1 × 10^4^ hPASMC were placed in each well of 6‐well plates and either NCM, IgG‐HCM, or EGFnAb‐HCM were placed on the cells. The hPASMC were incubated in normoxia for 48 h and viable cell numbers were determined by trypan blue exclusion. Incubating hPASMC with IgG‐HCM resulted in a near doubling of viable cell numbers compared to hPASMC incubated in NCM, and the HCM‐induced increase in viable cell number was prevented when the hPASMC were incubated with EGFnAb‐HCM (Figure 2c).

### 3.3 The role of EGFR in the HCM‐induced increase in Arg2 protein expression and viable cell numbers in hPASMC

**FIGURE 3:**
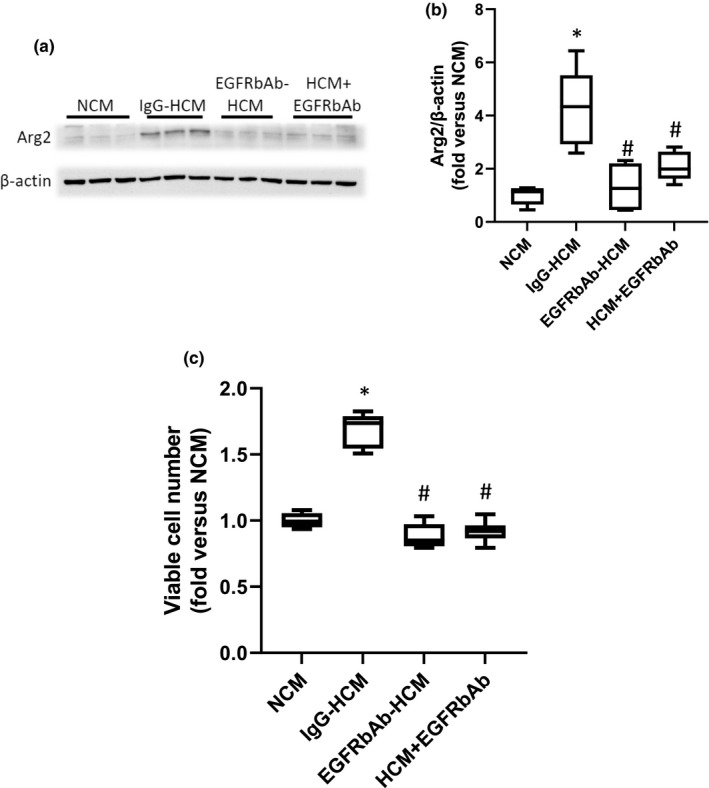
An EGFR blocking antibody prevented the HCM‐induced increase in hPASMC Arg2 protein and viable cell numbers. Either an IgG control (IgG‐HCM) antibody or an antibody against EGFR (EGFRbAb‐HCM) was added to the media during the generation of hypoxic conditioned media. NCM was generated as described in Figure 1. An additional set of hPASMC were treated with HCM that had EGFR blocking antibody added to it just prior to placing on the hPASMC (HCM + EGFRbAb) The NCM, IgG‐HCM, EGFRbAb‐HCM, or HCM + EGFRbAb was then placed on hPASMC with smooth muscle media in a 50:50 ratio and the hPASMC were incubated in room air with 5% CO_2_ for 24 h. (a) Protein was harvested for western blotting for Arg2 and β‐actin. Representative western blot images. (b) Arg2 protein levels were greater in hPASMC incubated with IgG‐HCM than in those incubated with NCM, EGFRbAb‐HCM, or HCM + EGFRbAb. Densitometry for the Arg2 western blots normalized to β‐actin. *Different from NCM *p* < 0.005. #Different from IgG‐HCM, *p* < 0.001. *n* = 6 for each group. (c) To measure viable hPASMC numbers, 1 × 10^4^ hPASMC were plated in each well of 6‐well plates, NCM, IgG‐HCM, EGFRbAb‐HCM, or HCM + EGFRbAb were placed on the cells, and after 48 h in normoxia viable cell numbers were determined using trypan blue exclusion. Viable hPASMC numbers were significantly greater after incubation with IgG‐HCM than after incubation with NCM, EGFRbAb‐HCM, or HCM + EGFRbAb. *Different from NCM *p* < 0.001, #Different from IgG‐HCM, *p* < 0.001. *n* = 6 for each group.

To determine the role of EGFR activation in the HMC‐induced increase in Arg2 protein expression and increase in viable cell numbers in hPASMC, we utilized an EGFR blocking antibody (EGFRbAb) added to the EC media during the generation of conditioned media. We generated hypoxic conditioned media from hPMVEC that were treated with either an isotype control antibody (IgG‐HCM) or EGFRbAb (EGFRbAb‐HCM). We also generated NCM from hPMVEC. Human PASMC were treated with either NCM, IgG‐HCM, or EGFRbAb‐HCM. Another set of hPASMC had the EGFRbAb added to the HCM immediately prior to placing on the hPASMC (HCM + EGFRbAb). The hPASMC in the four experimental groups were incubated in normoxia for 24 h and then the hPASMC were harvested for Arg2 western blotting. Treatment of hPASMC with EGFRbAb‐HCM prevented the HCM‐induced induction of Arg2 (Figure 3a,b). Similarly, adding EGFRbAb directly to HCM (HCM + EGFRbAb) immediately prior to placing on the hPASMC also prevented the HCM‐induced Arg2 protein expression (Figure 3a,b). To determine the effect of the EGFRbAb on viable cell numbers, 1 × 10^4^ hPASMC were placed in each well of 6‐well plates and either NCM, IgG‐HCM, EGFRbAb‐HCM, or HCM + EGFRbAb were placed on the cells. The cells were incubated in normoxia for 48 h and viable cell numbers were determined by trypan blue exclusion. The HCM‐induced increase in viable cell numbers was prevented when the hPASMC were incubated with either EGFRbAb‐HCM or when the EGFRbAb was added to the HCM (HCM + EGFRbAb) (Figure 3c).

### 3.4 hPMVEC‐derived HCM caused phosphorylation of hPASMC EGFR

**FIGURE 4:**
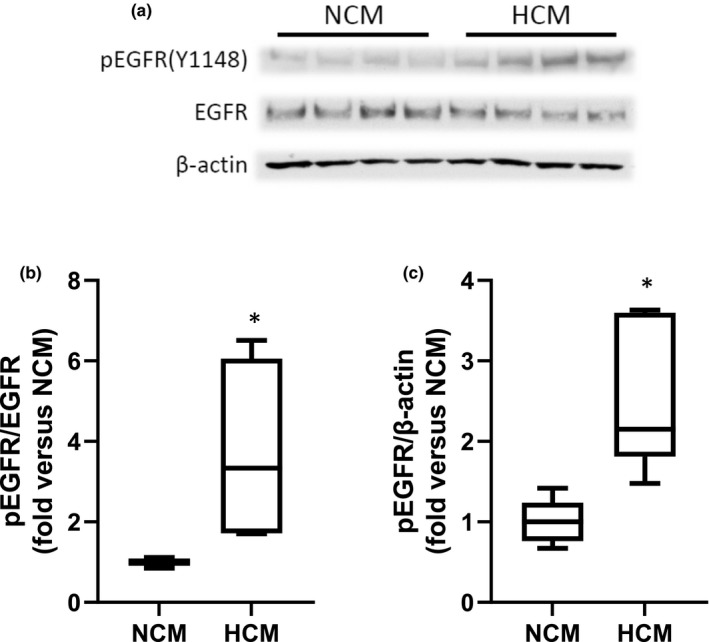
Hypoxic conditioned media from hPMVEC activated EGFR in hPASMC. hPASMC were serum‐starved overnight and then treated with NCM or HCM for 5 minutes. Cells were then harvested for western blots. (a) Representative western blots for phosphorylated EGFR (pEGFR), total EGFR, and β‐actin. (b) Densitometry for pEGFR normalized to total EGFR. HCM treated hPASMC had significantly greater levels of pEGFR than did NCM treated hPASMC. *HCM different from NCM, *p* < 0.05. *n* = 5 in each group. (c) Densitometry for pEGFR normalized to β‐actin. HCM treatment of hPASMC resulted in significantly greater levels of pEGFR than in NCM treated hPASMC. *HCM different from NCM, *p* < 0.01. *n* = 5 for each group.

To determine the effect of the hPMVEC‐derived HCM on EGFR activation in hPASMC, we utilized hPASMC that were serum‐starved for 24 h. NCM or HCM was placed on serum‐starved hPASMC for 5 min and protein harvested for western blotting for phosphorylated EGFR (pEGFR, Y1148). As shown in Figure 4a, the expression levels for pEGFR were greater in those hPASMC treated with HCM than in those treated with NCM. Densitometry results, normalized to either total EGFR (Figure 4b) or β‐actin (Figure 4c), demonstrated significantly greater induction of hPASMC pEGFR by HCM than in hPASMC incubated with NCM.

### 3.5 Knocking down hPASMC EGFR prevented the HCM‐induced increase in viable cell numbers

**FIGURE 5:**
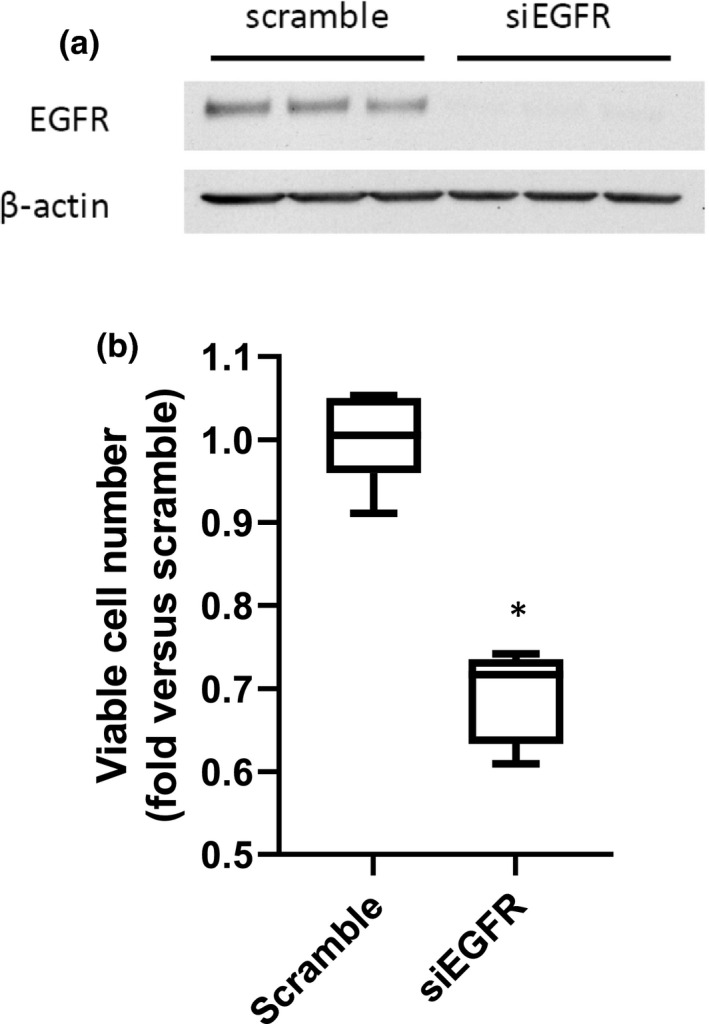
Treatment of hPASMC with an siRNA against EGFR prevented the HCM‐induced increase in hPASMC viable cell number. hPASMC were treated with either scramble siRNA or siRNA against EGFR (siEGFR) for 24 h, allowed to recover for 24 h, and then 1 × 10^4^ hPASMC were plated in each well of a 6‐well plate. The hPASMC were incubated in HCM in normoxia for 48 h. (a) Protein was isolated from the hPASMC and used in Western blotting for total EGFR followed by stripping and re‐probing with β‐actin. Representative Western blots for EGFR and β‐actin, demonstrating knockdown of EGFR by siEGFR. *n* = 3 for each group. (b) Viable cell numbers are determined by trypan blue exclusion. *HCM siEGFR different from HCM scramble, *p* < 0.001. *n* = 6 for each group.

To determine if preventing EGFR phosphorylation on hPASMC would prevent the HCM‐induced increase in viable cell numbers, we utilized siRNA‐mediated knockdown of EGFR expression in hPASMC. The hPASMC were transfected with siRNA against EGFR (siEGFR) or scramble siRNA for 24 h. The hPASMC were then allowed to recover for 24 h before treatment with HCM for 48 h for the determination of viable cell numbers. The siEGFR resulted in knockdown of EGFR in the hPASMC as evidenced by Western blotting (Figure 5a). The siEGFR‐transfected hPASMC had significantly fewer viable cells after incubation with HCM than did the scramble‐transfected hPASMC (Figure 5b).

### 3.6 Inhibiting hPASMC arginase prevented the HCM‐induced increase in viable hPASMC numbers

**FIGURE 6:**
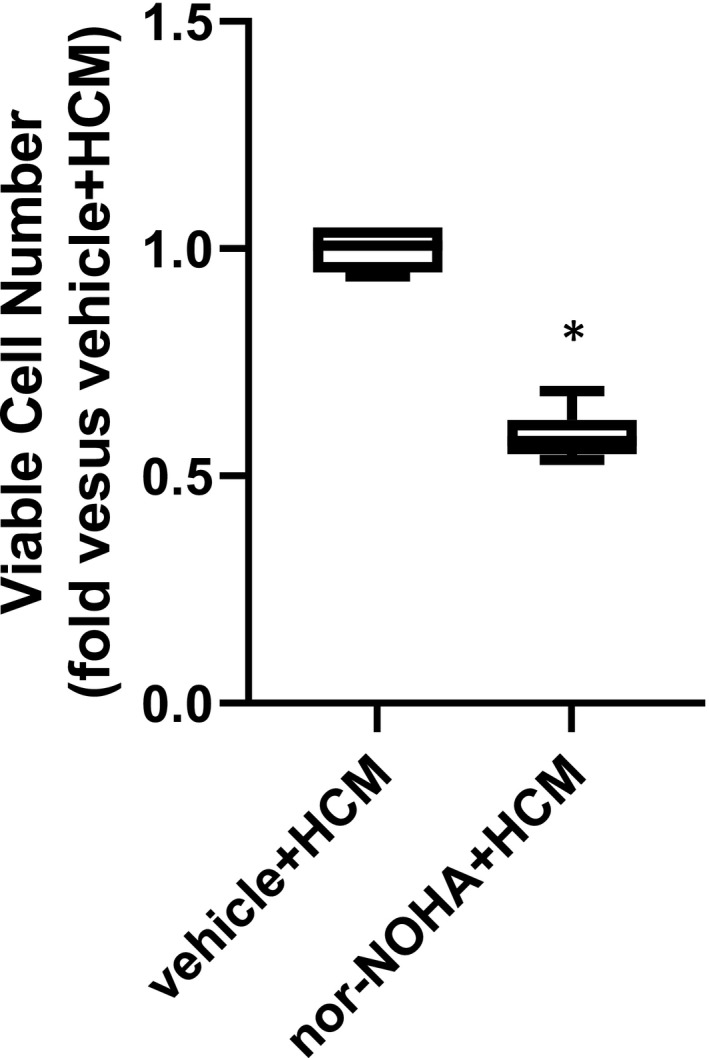
Treatment of hPASMC with an arginase inhibitor prevented the HCM‐induced increase in hPASMC viable cell numbers. Human PASMC (1 × 10^4^) were plated in each well of 6‐well plates and treated with either vehicle or 100 μM nor‐NOHA for 1 hour. HCM was then placed in the wells, the cells were incubated in normoxia and after 48 h the viable hPASMC numbers were counted using trypan blue exclusion. *Nor‐NOHA + HCM different from vehicle + HCM, *p* < 0.0001. *n* = 6 for each group.

To determine the role of hPASMC arginase in the HCM‐induced increase in viable hPASMC numbers, nor‐NOHA was used to inhibit arginase activity. HCM was generated as above. Human PASMC (5 × 10^4^) were seeded into each well of 6‐well plates. The hPASMC were treated with either vehicle or 100 μM nor‐NOHA (this dose was chosen based on our previous experience, see reference 5) 1 h prior to the addition of HCM. The hPASMC were then incubated in normoxia for 48 h and viable cell numbers were determined. Treatment with nor‐NOHA + HCM resulted in significantly fewer viable hPASMC than in hPASMC treated with vehicle + HCM (Figure 6).

## 4 DISCUSSION

**FIGURE 7:**
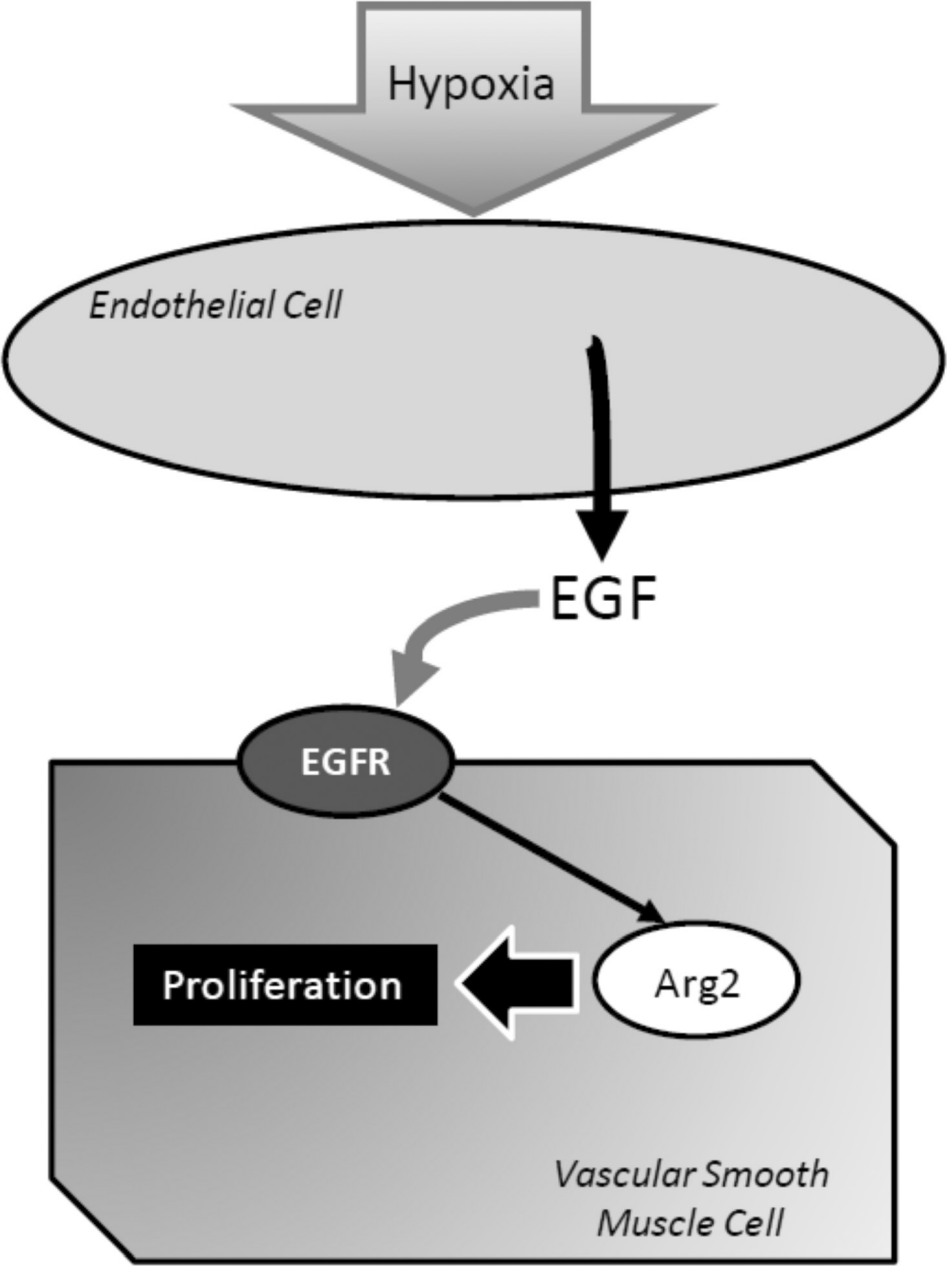
Proposed model. Hypoxia results in the release of EGF from pulmonary endothelial cells, which can then activate EGFR on the pulmonary vascular smooth muscle cells (SMCs) leading to induction of Arg2 which results in pulmonary vascular SMC proliferation. Antibodies against EGF or EGFR, or antagonists of Arg2, may be potential therapeutics in preventing hypoxia‐induced pulmonary vascular remodeling.

The main findings of this study were that pulmonary endothelial cells respond to hypoxia with the release of EGF, which resulted in the proliferation of pulmonary arterial SMCs via EGF‐EGFR‐mediated Arg2 up‐regulation. This interpretation is supported by the findings that in hPASMC incubated in normoxia HCM‐induced increases in Arg2 expression and viable cell numbers were prevented when: (1) The conditioned media was generated with an antibody against EGF included (EGFnAb‐HCM); or (2) an antibody against EGFR was added to the HCM (EGFRbAb‐HCM and HCM + EGFRAb). Additional evidence supporting our interpretation of the data comes from the findings that: (1) HCM induced the phosphorylation of EGFR on hPASMC; and (2) either knocking down EGFR in hPASMC or treating hPASMC with a small molecule inhibitor of arginase prevented the HCM‐induced increases in viable hPASMC numbers. As shown in Figure 7 our results are consistent with a mechanism whereby hypoxia induces EGF release from an endothelial cell, which can then activate EGFR on a SMC to induce Arg2 protein expression, resulting in proliferation of the hPASMC as evidenced by substantial increases in viable cell numbers in our experiments. These results suggest that future studies using antibodies against either EGF or EGFR, or an antagonist of arginase, as potential therapies to prevent PH related to lung disease and/or hypoxia (WHO Group 3) should be considered.

It is well known that endothelial cells and vascular SMCs interact in pulmonary vessels via release of vasoactive substances, microvesicles, junctional proteins, etc (Gao et al., 2016). However, crosstalk between pulmonary endothelial cells and pulmonary arterial SMCs specifically resulting in pulmonary arterial SMC proliferation is less well studied although it has been reported. For example, Eddahibi et al. (2006) found that media from human pulmonary endothelial cells stimulated PASMC proliferation and the effect on proliferation was diminished by the addition of a serotonin inhibitor. Similarly, Dewachter et al. (2006) found that the media from pulmonary endothelial cells treated with angiopoietin‐1 induced marked proliferation in PASMC and the proliferation was attenuated by treatment with a serotonin inhibitor. Mercier et al. (2017) found that media from pulmonary endothelial cells obtained from chronic thromboembolic PH patients elicited marked PASMC proliferation. Recently, it has been shown that hypoxia‐induced Twist1‐dependent platelet‐derived growth factor B (PDGFB) production in human pulmonary arterial endothelial cells (hPAEC) and transfection of the endothelial cells with siRNA against Twist1 attenuated hypoxic conditioned media‐induced arterial smooth muscle proliferation (Mammoto et al., 2020). These studies taken together with our findings suggest that endothelial‐vascular smooth muscle crosstalk may be important in SMC proliferation, and that the mechanism responsible for the crosstalk leading to SMC proliferation may depend on the stimulus applied and/or the phenotype (i.e., arterial vs. microvascular) of the endothelial cells that are being stimulated.

There are limited data available on the role of EGF in pulmonary vascular remodeling secondary to hypoxia, although a role for EGF in cancer has been well established (Mitchell et al., 2018). We have previously shown that EGF is a critical factor released from hypoxic pulmonary endothelial cells by demonstrating that siRNA‐mediated EGF knockdown in the pulmonary ECs used to generate HCM prevented the HCM‐induced increases in Arg2 protein and viable cell numbers in hPASMC (Pool et al., 2018). In the present study, we provide further evidence that hypoxia results in release of EGF by showing that treatment of endothelial cells with an EGF neutralizing antibody during the generation of HCM prevented the subsequent HCM‐induced increases in Arg2 protein and viable cell numbers in hPASMC (Figure 2). This is also consistent with our previous report in hPMVEC that culture in hypoxia‐induced a robust induction of EGF mRNA, and that an EGF neutralizing antibody or siRNA transfection against EGF prevented the hypoxia‐induced endothelial cell Arg2 protein expression and increase in viable endothelial cell numbers (Pool et al., 2018). It has been shown that hPASMC respond to EGF treatment with a robust proliferative response (Schultz et al., 2006; Tu et al., 2012). We have also reported in HeLa cells that hypoxia‐induced EGF mRNA expression and that an EGF neutralizing antibody inhibited the hypoxia‐induced increase in HeLa cell proliferation (Setty et al., 2017). Thus, taken together these data are consistent with EGF being the factor released from hypoxic pulmonary endothelial cells in our experiments that caused the proliferation in pulmonary arterial SMCs.

The role of EGFR activation has been well studied in cancer angiogenesis and proliferation of cancer cells, and EGFR antagonists and/or blocking antibodies are used to treat, or are being studied as treatments for, certain types of cancers (Agustoni et al., 2019; Santos et al., 2021). The notion that EGFR activation was key in the HCM‐induced Arg2 induction and proliferation in hPASMC in the present study is supported by four experiments. First, adding an antibody against EGFR while generating the HCM (EGFRbAb‐HCM) prevented the HCM‐induced increases in Arg2 protein and viable cell numbers in hPASMC (Figure 3). Second, adding an EGFR blocking antibody to the HCM (HCM + EGFRbAb) immediately prior to placing on the hPASMC also prevented the HCM‐induced increases in Arg2 protein and viable cell numbers (Figure 3). Third, we demonstrated that HCM led to EGFR phosphorylation in hPASMC (Figure 4a,b). Finally, we found that treating hPASMC with siRNA against EGFR attenuated HCM‐induced proliferation (Figure 4d). Activation of EGFR occurs through ligand binding, which causes dimerization of EGFR resulting in *trans*‐phosphorylation that enables interactions with cell signaling molecules (Fuller et al., 2008; Kovacs et al., 2015). An alternative model has been proposed wherein EGFR is a pre‐formed dimer at the cell surface and upon ligand binding “rotates” to allow for phosphorylation (Purba et al., 2017). Regardless, of which model of EGFR activation is occurring in the hPASMC it is clear that ligand binding is involved in the HCM‐mediated activation of EGFR in our studies. We have previously demonstrated in hPMVEC and HeLa cells that exposing the cells to hypoxia (1% O_2_) led to robust phosphorylation of EGFR (Pool et al., 2018; Setty et al., 2017). In this study, we found that HCM induced hPASMC EGFR phosphorylation and this HCM‐induced activation of EGFR on the hPASMC were necessary for the HCM‐induced Arg2 protein expression and proliferation. Although, in one early study EGFR inhibitors were shown to result in only limited attenuation of chronic hypoxia‐induced PH indices in mice (Dahal et al., 2010), more recent data have suggested a role for EGFR inhibitors in PH. For example, Norton et al. (2020) found attenuated chronic hypoxia‐induced pulmonary vascular remodeling in rats treated with the EGFR tyrosine kinase inhibitor Gefitinib. Similarly, Yu et al. (2019) found that the EGFR inhibitor dacomitinib prevented the chronic hypoxia‐induced increase in right ventricular systolic pressure and vascular remodeling in rats. The reasons for the disparate findings are unclear, although it may be due to species and/or PH model differences. Given the variable effects of EGFR inhibitors in PH, further studies are needed to fully elucidate the role of the EGF‐EGFR‐mediated pathway in chronic hypoxia‐induced pulmonary vascular diseases.

There are of course some important limitations of our study that should be considered. First, we studied pulmonary arterial SMCs in culture which may limit the applicability of our findings to the *in vivo* setting, particularly with reference to clinical disease. In pulmonary arterial SMCs in the medial layer of the vessel there are likely several phenotypes including a proliferative and a contractile phenotype (Frid et al., 1997; Lechartier et al., 2022), and in cell culture the SMCs proliferative phenotype tends to dominate (Lechartier et al., 2022; Thyberg, 1996). Although we did find dramatic differences in Arg2 expression and proliferation in cultured SMCs depending on whether the conditioned media came from normoxic or hypoxic microvascular endothelial cells. Second, our results in hPASMC are similar to our findings in hPMVEC, which given the ability of endothelial cells to go through endothelial‐mesenchymal transition (Díez et al., 2010; Lechartier et al., 2022) could represent aspects of a common embryologic lineage for pulmonary endothelial and pulmonary vascular endothelial cells (Hall et al., 2000). Future studies in the more complex *in vivo* setting are needed to further define the role of endothelial cell EGF release in the vascular remodeling associated with hypoxia‐induced PH.

In conclusion, hypoxia exposed hPMVEC released EGF which activated EGFR on hPASMC resulting in hPASMC up‐regulation of Arg2 and increased viable hPASMC numbers. These results extend our previous work in hPMVEC (Pool et al., 2018) and demonstrate a role for pulmonary endothelial cells in the hypoxia‐induced proliferation of pulmonary arterial SMCs. This endothelial cell‐SMC interaction via the EGF‐EGFR pathway may contribute to vascular remodeling, particularly in hypoxia‐induced PH (WHO Class 3). Furthermore, these studies suggest that preventing activation of EGFR on pulmonary arterial SMCs using EGF and/or EGFR antibodies may be potential therapeutic modalities to prevent or treat hypoxia‐induced pulmonary vascular remodeling.

## AUTHOR CONTRIBUTIONS

Dr. Bernadette Chen contributed to conception of the work, analysis, and interpretation of data, and drafting and revising for intellectual content. Dr. Yi Jin contributed to design of the work, acquisition and analysis of the data, and drafting the work. Caitlyn Pool contributed to the acquisition and analysis of the data and revising for intellectual content. Dr. Yusen Liu contributed to design of the work, analysis and interpretation of data, and revising for intellectual content. Dr. Leif Nelin contributed to design of the work, analysis and interpretation of data, and revising for intellectual content. All authors gave final approval of the version to be published and agree to be accountable for all aspects of the work in ensuring that questions related to accuracy are appropriately investigated and resolved.

## CONFLICT OF INTEREST

The authors have no conflicts of interest to disclose.
